# Determinants of Exclusive Breastfeeding and Mixed Feeding Among Mothers of Infants in Dubai and Sharjah, United Arab Emirates

**DOI:** 10.3389/fnut.2022.872217

**Published:** 2022-05-10

**Authors:** Haleama Al Sabbah, Enas A. Assaf, Zainab Taha, Radwan Qasrawi, Hadia Radwan

**Affiliations:** ^1^Department of Health Sciences, Zayed University, Dubai, United Arab Emirates; ^2^Department of Community Nursing, Faculty of Nursing, Applied Science Private University, Amman, Jordan; ^3^Department of Health Sciences, Zayed University, Abu Dhabi, United Arab Emirates; ^4^Department of Computer Science, Al-Quds University, Jerusalem, Palestine; ^5^Department of Computer Engineering, Istinye University, Istanbul, Turkey; ^6^Department of Clinical Nutrition and Dietetics, College of Health Sciences, Research Institute of Medical and Health Sciences, University of Sharjah, Sharjah, United Arab Emirates

**Keywords:** exclusive breastfeeding, mixed feeding, practices, Sharjah, Dubai, UAE, child under two years

## Abstract

**Background:**

Breastfeeding (BF) is considered the ultimate method of infant feeding for at least the first 6 months of life. Exclusive breastfeeding (EBF) is one of the most effective interventions to improve child survival. The main objective of this study was to assess the prevalence and duration of exclusive breastfeeding and the associated factors among women in Dubai and Sharjah, UAE.

**Methods:**

A cross-sectional study was conducted in four hospitals and four healthcare centers in Dubai and Sharjah between September 2017 and December 2017. Hospitals and centers are governmental and provide maternal and child health services. A convenience sample of 858 Arab and Emirati mothers with children under the age of 2 years participated in the study. Face-to-face interviews were conducted by using structured questionnaires. The study was approved by the University Ethical Committee and the UAE Ministry of Health before data collection. Descriptive statistics were computed to describe all the questionnaire items. The chi-square test was used to compare the study's categorical variables. A binary logistic regression analysis was used to predict the relationship between BF and its associated factors. Statistical tests with *P*-values < 0.05 were considered statistically significant.

**Results:**

The mean age of the participating mothers was 30.6 (SD 5.5) years. Results showed that the prevalence of exclusive breastfeeding among the study participants was 24.4% (31.1% in Sharjah and 22% in Dubai; *P* = 0.003). The binary logistic regression reported that mother's and father's education, skin-to-skin period, number of children, mothers' health, and place of living were significantly associated with exclusive breastfeeding (*P* < 0.05). The results reported a significant association between EB and duration of breastfeeding (OR = 6.9, *P* = 0.002), husband education (OR = 2.1, *P* = 0.015), mother education (OR = 1.3, *P* = 0.027), number of children (OR = 7.9, *P* = 0.045), having any health problem (OR = 1.2, *P* = 0.045), and living place (OR = 1.4, *P* = 0.033), and a non-significant positive effect of family size and family income. Furthermore, the result reported a significant association between mixed breastfeeding and duration of breastfeeding (OR = 0.1, *P* = 0.000), skin-to-skin period (OR = 0.3, *P* = 0.002), underweight (OR = 4.7, *P* = 0.034), last infant's sex (OR = 1.6, *P* = 0.010), having maid at home (OR = 2.1, *P* = 0.000), number of children (OR = 0.2, *P* = 0.013), and living place (OR =1.1, *P* = 0.014), and a non-significant association with family size and family income.

**Conclusions:**

Therefore, a health promotion program for exclusive breastfeeding during antenatal health visits, together with initiating health policies in maternal hospitals to encourage the initiation of breastfeeding during the first hour of birth and the introduction of skin-to-skin contact during the first 5 min of birth are highly recommended.

## Introduction

Appropriate feeding practices during infancy and early childhood are essential to meet children's nutritional requirements and to maintain healthy growth and development (1). Substantial evidence supports breastfeeding as the best method for feeding infants and young children, providing them with optimal health and development (2). Accordingly, the World Health Organization (WHO) and the United Nations Children's Funds (UNICEF) recommend that breastfeeding should be initiated early within 1 h of birth, and to continue exclusive breastfeeding with no other foods or liquids for the first 6 months of life (3). This is followed by the introduction of complementary feeding and continued breastfeeding until at least 24 months of age (4).

The benefits of breastfeeding (BF) have been well documented, with solid evidence supporting its impact on reducing the prevalence of both mild and moderate malnutrition as well as childhood diseases (5). Long-term benefits have also been established for BF in terms of the prevention of diseases such as obesity, heart disease, diabetes, and asthma (6–9). However, the WHO and UNICEF have pointed out that the benefits of BF would be achieved when mothers breastfeed their babies for the first 6 months exclusively, i.e., only breast milk (3). Infants who receive any BF would benefit from the nutrients in breast milk and other advantages of BF, such as bonding, cognitive development, and enhancement of the immune system (2). The protective effect from obesity and other childhood diseases on infants and young children fed breast milk might be enhanced through its effect on infant microbiota colonization and development (10, 11).

Breastfeeding directly affects the infant's gut microbiota by exposure to the milk microbiota and indirectly *via* maternal milk factors that affect bacterial growth and metabolism, such as human milk oligosaccharides, secretory IgA, and antimicrobial factors. The potential of breast milk is important in protecting infants from asthma and allergies (12). Among the important core stone benefits of breastfeeding is that it improves child survival in the face of highly infectious diseases like COVID-19 (13). The WHO recommends that breastfeeding should not be discontinued in cases of suspected or being confirmed COVID-19 (13). Thus, the benefit of breastfeeding can overcome the risk of catching the infection as infants acquire passive IgA immunity. This outweighs the potential COVID-19 risks (14).

Despite considerable efforts to promote breastfeeding practices, the Gulf region is still behind when it comes to the goals set by the WHO (15). A study examining breastfeeding practices in the Middle East revealed that a large number of mothers supplemented breastfeeding with other forms of feeding at an early age (16, 17). Since infant nutrition and health are interrelated, the effects of breastfeeding and maternal nutrition on each of these outcomes should be addressed. Diet is an important environmental factor that may influence the health outcomes of breastfeeding mothers and infants. The maternal diet may affect the formation, composition, or secretion of milk. Studies have shown that unhealthy diets and food allergies play a role in the development of asthma in the Gulf countries (18). This finding is of great significance, considering the high prevalence of asthma among children and adults in Gulf countries, to the extent of becoming a significant public health concern.

Similarly, researchers from different countries in Europe have found that breastfeeding practices do not meet the WHO and UNICEF recommendations (19). They pointed out that exclusive breastfeeding practices in different countries in Europe do not meet the 2025 World Health Assembly's Global Target for Nutrition to increase the rate to at least 50% (20). As for the UAE, mixed feeding, complimentary food, and fluid additions have been introduced in the first month of life in the UAE (21). Several factors negatively affect breastfeeding practices in different Gulf countries, such as maternal age, level of education, mothers' perception of insufficient milk production, problems associated with the breast such as nipple problems, mode of delivery (cesarean section), and hospital practices such as non-rooming-in (22, 23). In these studies, a high educational level was more strongly associated with lower BF initiation and exclusive breastfeeding rates. In addition, hospital practices played an important role in breastfeeding outcomes, where vaginal birth and rooming enhanced breastfeeding initiation and extended the breastfeeding duration. In addition, mothers' perception of insufficient milk production and problems associated with the breast, such as nipple problems, have been reported to reduce the rate and duration of exclusive BF. The factors contributing to the continuation of breastfeeding and mixed feeding vary from country to country (24–28). One study in Malawi found that ethnicity of the mother, younger age of the mother, female infant, and high number of children were significantly associated with EBF practices (29). While a study among Cambodian mothers found that those with middle wealth were less likely to go for EBF compared to low wealth mothers (30). Another study among Irish mothers found that maternal age, short maternity leave, mothers from Irish nationality, non-tertiary education, and neonates with intensive-care unit admission were more likely not to adhere to EBF compared to others (31). While concerning Irish primigravida mothers' non-adherence to EBF, the study found that mothers' higher body mass index, unemployment, gestational diabetes, low-birth-weight antenatal steroids, and hypernatremia were all highly associated factors (32). Whereas, reasons for the discontinuation of breastfeeding might include maternal age, educational background, socioeconomic status, postpartum depression, maternal confidence, maternal obesity, and being overweight (33). On the other hand, factors associated with a higher breastfeeding rate and longer duration include increased maternal age, low educational levels, rural residence, low income, multiparity, and avoiding contraceptives (34).

To maintain breastfeeding as the best feeding method that supports infants and young children's health, the WHO has set a global goal to increase the rate of exclusive breastfeeding to at least 50% by 2025 (35). The Ministry of Health in the UAE has made extensive efforts in collaboration with health authorities in all emirates to develop plans and strategies that would help achieve this goal by increasing the rate of EBF (36). As part of these efforts, the UAE has embraced various policy initiatives, including the Baby-Friendly Hospital Initiative (BFHI), the Global Strategy for Infant and Young Child Feeding, and the implementation of the International Code of Marketing of Breast Milk Substitutes (37). According to the MOH national infant feeding policy implemented throughout the country, infants should be breastfed exclusively until 6 months of age (21, 38).

In addition, the UAE Federal National Council passed a draft clause in the child rights law to make breastfeeding mandatory for the first 2 years of an infant's life (39). To support BF among working mothers, a decree was issued that extended the 60 days of paid maternity leave to 90 days. The experience of the Emirate of Sharjah is peculiar, as the city has been recognized as the Middle East's first baby-friendly city following the successful adoption of the main standards for this rating (40). To assess these efforts, it is important to assess breastfeeding practices and determine the EBF rates. Few studies have been conducted on breastfeeding in the UAE and recent studies have been confined to certain emirates. Most of these studies were cross-sectional and only one national survey was conducted in the year 2000. The results of the national survey revealed that only 34% of the infants were exclusively breastfed for up to 4 months of age (36). A recent study conducted in Abu Dhabi reported a rate of 44.3% (41).

Despite the tremendous efforts to increase breastfeeding worldwide, the rates are suboptimal in many countries, including the UAE. In the UAE, there are gaps in understanding why many mothers have difficulties initiating and maintaining exclusive breastfeeding in the first 6 months of life and instead introduce artificial feeding. Therefore, in light of the limited success in EBF promotion, as evidenced by low EBF rates, there are factors affecting infant feeding and breastfeeding practices. Hence, in the current study, exploring these difficulties and associated factors can be amended through education programs and directing governmental intervention efforts to increase the rate of exclusive breastfeeding and meet the WHO and UNICEF goals. In addition, there are ongoing national efforts and investments in these programs, including the development and updating of policies and strategies. However, regardless of the health authorities' efforts to support and promote breastfeeding, the rate of exclusive breastfeeding in the UAE remains suboptimal. Therefore, this study will help assess the prevalence of exclusive breastfeeding and identify other associated factors that impact the duration of exclusive breastfeeding in infants aged 6–24 months in Dubai and Sharjah. Ultimately, the results can help health providers improve mothers' knowledge about breastfeeding. Furthermore, identifying these factors will shed light on why the breastfeeding rates are still suboptimal. Accordingly, there is very little documentation of EBF in the UAE due to the rapid changes in women's lifestyles and engagement in the workforce particularly in Dubai. The main objective of this study was to assess the prevalence of exclusive breastfeeding and to identify the main contributing factors in infants aged 6–24 months in Dubai and Sharjah to improve the public's knowledge and initiate health policies about breastfeeding.

## Materials and Methods

### Study Design and Settings

A cross-sectional study design was used to collect data from the waiting areas of the largest maternal and child outpatient clinics in four hospitals and four health centers in Sharjah and Dubai, UAE.

Data from a convenience sample of 858 mothers were collected between September 2017 and December 2017. Permission and ethical approval to conduct this study was obtained from the University Ethical Committee and the UAE Ministry of Health. Written informed consent was obtained from mothers who met the criteria for this study and were willing to participate. Participants were informed that their participation in this study was voluntary and that they had the freedom to quit the study at any time.

### Population and Sampling

To be included in the study, mothers had to be aged 18 years and above, be either Emiratis or Arabs, be able to provide written consent, and have at least one child aged 6 months to 2 years. Participants were excluded if they were <18 years old or had children aged <6 months or more than 2 years. The proposed sample size was to collect data from at least 800 women in waiting areas (100 women from each clinic/center). A total of 858 women (492 living in Dubai and 366 living in Sharjah) participated in this study.

### Data Collection

Data were collected using eight trained interviewer-administered multicomponent questionnaires through a structured face-to-face interview at the selected outpatient clinic waiting rooms in hospitals and healthcare centers in Dubai and Sharjah. The research assistant approached mothers visiting outpatient clinics in the waiting rooms of hospitals and public health centers in Dubai and Sharjah and introduced the study with its objectives and protocol. Eligible and interested subjects read and signed a consent form prior to starting face-to-face interviews. A multicomponent questionnaire was developed based on a literature review of similar studies and was reviewed by a panel of experts in the field of infant feeding (42–44). A valid and reliable questionnaire was used to collect the data.

The structured interview questionnaire was translated into Arabic, then back-translated into English, and pilot tested with 66 mothers from one of the hospitals' outpatient clinics in Dubai (the results from the pilot study were not included in this study and were only used for piloting) to ensure the clarity, simplicity, and logical flow of the questions. The questionnaire was revised according to the pilot study. The final version of the questionnaire consisted of 49 questions and required approximately 10–15 min to complete.

The questionnaire consisted of four main sections: sociodemographic data about the mother and the child (17 items; e.g., maternal age, maternal marriage age, mother and father educational level, place of living, maternal employment, family size, number of children, income, having a maid, infant age, birth weight, etc.); family socioeconomic status (five items); knowledge, attitude, and practice of breastfeeding and complementary feeding (27 items); and the mother's obstetric and general health status section (eight items; e.g., type of delivery, lactation amenorrhea, use of contraception, if she is currently pregnant, sore nipples, maternal health perception, and complications), and breastfeeding practices such as (initiation time of breastfeeding, skin-to-skin care duration, breastfeeding duration, infant feeding type).

### Anthropometric Measurements

Infant birth weight and length were obtained from the children's health cards, while the mother's weight (kg) and height (cm) were measured during the visit using a standard protocol using the Seca 220 Telescopic Measuring Rod for Column Scales for height/weight measurements. The BMI (kg/m^2^) was calculated by dividing the weight (kg) by the height squared (m). The BMI was determined according to the World Health Organization (WHO) classification (45).

### Breastfeeding Outcomes

Early initiation of breastfeeding was defined as the proportion of children who latched the breasts within 1 h of birth. Exclusive breastfeeding was defined as an infant fed only breast milk without any other oral intake, except for medications and vitamins, within the last 24 h. Mixed feeding was defined as the introduction of solid food or formula milk during breastfeeding.

Formula feeding was defined as feeding only formula from birth.

### Statistical Analysis

Data were entered, cleaned, and analyzed using the Statistical Package for Social Science (SPSS) software version 24. Descriptive statistics were computed to describe all the questionnaire items including frequencies and percentages. Furthermore, inferential statistical analysis, including the chi-square test, ANOVA test, and binary logistic regression analysis (OR) were used to assess the relationships between BF and its associated factors (infant's age, duration of BF, skin-to-skin period, parental education, last infant's sex, maid at the house, birth weight, number of children, health problems, marriage age of mother, family size, family income, and Emirate states). The significant level was set at *P* < 0.05.

## Results

The mean age of the participant mothers was 30.6 (SD, 5.5) years. Table 1 presents the demographic characteristics of women, as about 44% were from Sharjah and the rest were from the Dubai Emirate. Approximately two-thirds of the women were aged between 20 and 34 years. Most women had more than a high school education and were married at the time of the data collection. Only 29% of the women were working, and about two-thirds of the women reported being in the upper- and middle-income groups.

**Table 1:** Sociodemographic characteristics of participating women in both Dubai and Sharjah.

**Sociodemographic characteristics**		**Emirate (*****n*** **=** **858)**					
		**Sharjah**		**Dubai**		**Total**	
		***n***	**%**	***n***	**%**	***n***	**%**
Mother education	High school and less	107	26.7	168	34.1	275	30.8
	Higher than high school	294	73.3	325	65.9	619	69.2
Mother occupation	Working	83	20.7	180	36.5	263	29.4
	Not working	318	79.3	313	63.5	631	70.6
Husband education	High school and less	89	22.2	152	30.8	241	27.0
	Higher than high school	312	77.8	341	69.2	653	73.0
Husband's working status	Working fulltime	399	99.5	480	97.4	879	98.3
	Not working	2	0.5	13	2.6	15	1.7
Family income	Middle and lower income	187	57.7	69	15.2	256	32.9
	Upper than middle income	137	42.3	386	84.8	523	67.1
Family size	≤ 4	214	57.7	237	50.6	451	53.8
	>4	157	42.3	231	49.4	388	46.2
Number of children	<3	248	61.8	290	58.8	538	60.2
	≥3	153	38.2	203	41.2	356	39.8
Marital status	Married	399	99.5	473	95.9	872	97.5
	Divorced or widow	2	0.5	20	4.1	22	2.5
What is your last infant's sex?	Boys	222	55.4	254	51.5	476	53.2
	Girls	179	44.6	239	48.5	418	46.8
Age group	15–19	5	1.3	4	0.8	9	1.0
	20–34	307	77.5	350	74.3	657	75.8
	35–50	84	21.2	117	24.8	201	23.2
Infants age (month)	1–6	68	17.0	52	10.5	120	13.4
	7–12	171	42.6	176	35.7	347	38.8
	13–18	115	28.7	161	32.7	276	30.9
	≥19	47	11.7	104	21.1	151	16.9

**Figure 1:**
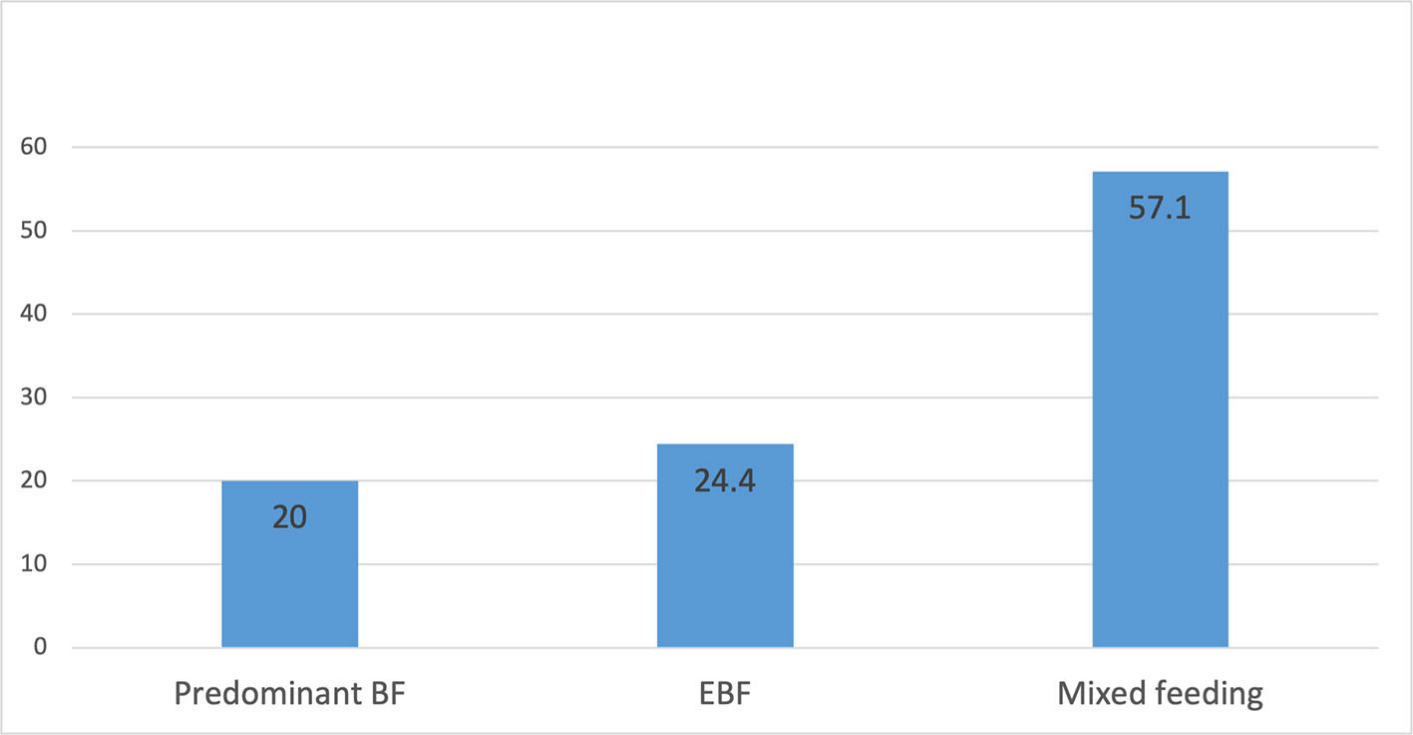
Prevalence breastfeeding practices among women (predominant breastfeeding, exclusive breastfeeding and mixed feeding).

Figure 1 shows the prevalence rates of different breastfeeding practices: EBF (24.4%), predominant breastfeeding (20.0%), and mixed feeding (57.1%).

Tables 2, 3 present the associations between EBF and mixed feeding (breast milk and formula milk), and the selected sociodemographic characteristics. Significant associations were found between the place of residence, employment, and EBF (*P* < 0.05). Participants living in the Emirate of Sharjah and non-working women had more EBF than those living in Dubai and working women (*P* = 0.003).

**Table 2:** Association between EBF and women's sociodemographic characteristics.

**Sociodemographic characteristics**		**Exclusive breastfeeding (*****n*** **=** **858)**						***P*** **-Value**
		**Yes**		**No**		**Total**		
		***n***	**%**	***n***	**%**	***n***	**%**	
Emirate	Sharjah	119	54.6	263	42.8	382	45.9	0.003^*^
	Dubai	99	45.4	351	57.2	450	54.1	
Marriage age of mother	≤ 20	75	35.0	175	28.6	250	30.3	0.079
	>20	139	65.0	436	71.4	575	69.7	
Marital status	Married	213	97.7	600	97.7	813	97.7	0.991
	Divorced or widow	5	2.3	14	2.3	19	2.3	
Mother education	High school and less	68	31.2	193	31.4	261	31.4	0.948
	Higher than high school	150	68.8	421	68.6	571	68.6	
Mother occupation	Working	41	18.8	199	32.4	240	28.8	0.000^*^
	Not working	177	81.2	415	67.6	592	71.2	
Husband education	High school and less	53	24.3	182	29.6	235	28.2	0.133
	Higher than high school	165	75.7	432	70.4	597	71.8	
Husband occupation	Working fulltime	217	99.5	603	98.2	820	98.6	0.156
	Not working	1	0.5	11	1.8	12	1.4	

* *P-value significant at P = < 0.005*.

**Table 3:** Association between mixed feeding (breast milk and formula milk and women's sociodemographic characteristics.

**Women characteristics**		**Mixed feeding (breast milk** **+** **formula milk) (*****n*** **=** **858)**						***P*** **-Value**
		**Yes**		**No**		**Total**		
		***n***	**%**	***n***	**%**	***n***	**%**	
Emirate	Sharjah	192	44.1	190	47.9	382	45.9	0.282
	Dubai	243	55.9	207	52.1	450	54.1	
Marriage age of mother	≤ 20	122	28.2	128	32.7	250	30.3	0.162
	>20	311	71.8	264	67.3	575	69.7	
Marital status	Married	428	98.4	385	97.0	813	97.7	0.173
	Divorced or Widow	7	1.6	12	3.0	19	2.3	
Mother education	High School and Less	128	29.4	133	33.5	261	31.4	0.206
	Higher than High School	307	70.6	264	66.5	571	68.6	
Mother occupation	Working	158	36.3	82	20.7	240	28.8	0.000^*^
	Not Working	277	63.7	315	79.3	592	71.2	
Having maid at home	Yes	226	69.1	173	42.2	435	57.1	0.000^*^
	No	117	30.9	210	54.8	372	42.9	
Husband occupation	Working fulltime	428	98.4	392	98.7	820	98.6	0.673
	Not working	7	1.6	5	1.3	12	1.4	

* *P-value significant at P = < 0.005*.

The results showed that working women and those who had a maid at home were significantly associated with mixed feeding (*P* < 0.001; Table 3).

Table 4 shows the associations between EBF and a mother's obstetric and general health status variables as type of delivery methods; being pregnant; use of contraceptives; complaints of sore nipple; maternal overall health status, and body mass index (BMI) were not significantly associated with EBF (*P* = 0.796, 0.192, 0.409, 0.364, 0.192), respectively. Only lactational amenorrhea during breastfeeding was significantly associated with EBF *(P* < 0.001).

**Table 4:** Association between EBF and women's obstetric and general health status variables.

**Women's overall health status**		**Exclusive breastfeeding (*****n*** **=** **858)**						***P*** **-Value**
		**Yes**		**No**		**Total**		
		***n***	**%**	***n***	**%**	***n***	**%**	
Delivery mode	Normal	154	70.6	428	69.7	582	70.0	0.796
	Cesarean	64	29.4	186	30.3	250	30.0	
Pregnancy status	Yes	27	12.4	57	9.3	84	10.1	0.192
	No	191	87.6	557	90.7	748	89.9	
Do you have any health problems?	Yes	25	11.5	89	14.5	114	13.7	0.264
	No	193	88.5	525	85.5	718	86.3	
Contraceptive use	Yes	52	23.9	164	26.7	216	26.0	0.409
	No	166	76.1	450	73.3	616	74.0	
Sore nipples	Yes	76	34.9	234	38.1	310	37.3	0.364
	No	142	65.1	380	61.9	522	62.7	
lactation amenorrhea	Yes	138	63.3	267	43.5	405	48.7	0.000
	No	80	36.7	347	56.5	427	51.3	
Mother BMI	Underweight (<18.5)	3	1.5	11	1.9	14	1.8	0.078
	Normal (18.5–25)	93	46.5	213	37.2	306	39.6	
	Overweight (26–29)	69	34.5	208	36.3	277	35.8	
	Obese (≥30)	35	17.5	141	24.6	176	22.8	

^*^P value significant at P = < 0.001.

Regarding mixed feeding and women's overall health status, as shown in Table 5, those who used contraceptives were more likely to use mixed feeding than those who did not (*P* < 0.010). In addition, women who had amenorrhea while breastfeeding were more likely not to use mixed feeding than those who did not (*P* < 0.000).

**Table 5:** Association between mixed feeding (breast milk and formula milk) and women's health status.

**Women's health characteristics**		**Mixed feeding (breast milk** **+** **formula milk) (*****n*** **=** **858)**						***P*** **-Value**
		**Yes**		**No**		**Total**		
		***n***	**%**	***n***	**%**	***n***	**%**	
Type of delivery	Normal	294	67.6	288	72.5	582	70.0	0.119
	Cesarean	141	32.4	109	27.5	250	30.0	
Pregnancy status	Yes	41	9.4	43	10.8	84	10.1	0.509
	No	394	90.6	354	89.2	748	89.9	
Do you have any health problems?	Yes	64	14.7	50	12.6	114	13.7	0.375
	No	371	85.3	347	87.4	718	86.3	
Contraceptive use	Yes	130	29.9	86	21.7	216	26.0	0.007
	No	305	70.1	311	78.3	616	74.0	
Sore nipples	Yes	167	38.4	143	36.0	310	37.3	0.480
	No	268	61.6	254	64.0	522	62.7	
lactational amenorrhea	Yes	165	37.9	240	60.5	405	48.7	0.000
	No	270	62.1	157	39.5	427	51.3	
Mother BMI	Under weight (<18.5)	7	1.7	7	1.9	14	1.8	0.515
	Normal (18.5–25)	160	38.6	146	40.7	306	39.6	
	Over Weight (26–29)	144	34.8	133	37.0	277	35.8	
	Obese (≥30)	103	24.9	73	20.3	176	22.8	

^*^P value significant at P = < 0.005.

Table 6 shows that women who started breastfeeding soon after delivery in <1 h were significantly associated with EBF (*P* < 0.010). In addition, a longer breastfeeding period was significantly associated with EBF (*P* < 0.000).

**Table 6:** Association between EBF and different feeding practices.

**Women's breastfeeding practices**		**Exclusive breastfeeding (*****n*** **=** **858)**						***P*** **-Value**
		**Yes**		**No**		**Total**		
		***n***	**%**	***n***	**%**	***n***	**%**	
Breastfeeding during the 1^st^ hour after birth	Yes	174	79.8	429	69.9	603	72.5	0.005
	No	44	20.2	185	30.1	229	27.5	
When did you start breastfeeding?	Directly after delivery (within the first hour)	174	79.8	429	69.9	603	72.5	0.007
	After one hour	35	16.1	113	18.4	148	17.8	
	After 1 day	5	2.3	40	6.5	45	5.4	
	After few days	4	1.8	32	5.2	36	4.3	
Infant still breastfeeding	Yes	124	56.9	211	34.4	335	40.3	0.000
	No	94	43.1	402	65.5	496	59.6	
Breastfeeding duration	≤ 6months	37	38.9	271	65.9	308	60.9	0.000
	>6months	58	61.1	140	34.1	198	39.1	
Skin to skin contact period	≤ 5 min	75	43.9	188	42.0	263	42.5	0.661
	6–10 min	31	18.1	96	21.4	127	20.5	
	>10	65	38.0	164	36.6	229	37.0	

*P-value significant at P = < 0.005.

The results of the logistic regression in Table 7 indicate that exclusive breastfeeding and mixed breastfeeding of mothers living in the UAE are affected by many factors. The determinants of breastfeeding indicated by “exclusive and mixed formula breastfeeding” among Emirate mothers are in the overall population, before and during the pandemic, is assessed by several variables of which duration of breastfeeding, skin-to-skin period, having made at home, having any health problem, family income, gender, infant age, family size, and family income.

**Table 7:** Odds ratio between EBF and mixed feeding together with some variables.

**Variables**		**Exclusive breastfeeding**				**Mixed breastfeeding**			
		**OR**	**CI 95%**		***P*** **-Value**	**OR**	**CI 95%**		***P*** **-Value**
Infants age (month)	1–6	1.0				1.0			
	7–12	1.1	0.3	4.5	0.361	1.2	0.4	3.8	0.297
	13–18	0.6	0.1	2.5	0.397	1.8	0.5	6.2	0.325
	≥19	0.8	0.1	4.2	0.898	2.7	0.6	11.5	0.755
Duration of breastfeeding	0–6 months	1.0				1			
	7–12 months	2.0	0.8	4.7	0.051	0.2	0.1	0.5	0.000
	≥13 months	6.9	2.0	23.8	0.002	0.1	0.0	0.2	0.000
Skin to skin period	≤ 5 min	1.0				1			
	6–10 min	0.8	0.3	2.3	0.066	0.7	0.3	1.7	0.234
	>10	2.0	0.9	4.5	0.042	0.3	0.2	0.6	0.002
Underweight	No	1.0				1			
	Yes	0.7	0.1	7.2	0.722	4.7	0.4	50.2	0.034
Husband education	High school and less	1.0				1			
	Higher than high school	2.1	0.8	5.5	0.015	0.6	0.3	1.2	0.241
Mother education	High school and less	1.0				1			
	Higher than high school	1.3	0.5	3.5	0.027	1.4	0.6	3.1	0.180
What is your last infant's sex?	Girls	1.0				1			
	Boys	0.7	0.3	1.4	0.180	1.6	0.9	2.9	0.010
Do you have a maid at home?	No	1.0				1			
	Yes	0.8	0.4	1.6	0.072	2.1	1.1	4.0	0.000
Birth weight	Normal birth weight	1.0				1			
	Low birth weight	1.1	0.3	4.8	0.920	0.8	0.2	3.0	0.680
Number of children	<3	1.0				1			
	≥3	7.9	1.0	65.2	0.045	0.2	0.0	0.6	0.013
Do you have any health problem?	No	1.0				1			
	Yes	1.2	0.4	3.2	0.045	0.9	0.4	2.1	0.065
Marriage age of mother	≤ 20	1.0				1			
	>20	0.7	0.3	1.6	0.320	1.3	0.6	2.5	0.255
Family size	≤ 4	1.0				1			
	>4	0.1	0.0	1.1	0.271	7.1	1.8	28.3	0.926
Family income	Middle and lower income	1.0				1			
	Upper than Middle income	1.0	0.4	2.5	0.468	2.5	1.1	5.7	0.755
Emirate	Dubai	1.0				1			
	Sharjah	1.4	0.6	3.5	0.033	2.5	1.1	5.8	0.014

*P-value significant at P = < 0.050.

To explain exclusive breastfeeding, the odds ratio (OR) of women with infants' age >12 months are [0.6, 0.8 (95% C. I: (0.1–2.5), (0.1–4.2)); 1.8, 2.7 (95% C.I: (0.5–6.2), (0.6–11.5))] for EBF and mixed BF, respectively. Predictors (breastfeeding duration, skin-to-skin period, fathers' and mothers' educational levels, number of children, and place of residence) were significant for exclusive breastfeeding. For mixed breastfeeding, the predictors (duration of breastfeeding, skin-to-skin period, infant underweight, infant sex, maid at home, number of children, and living place) were significant with mixed BF variables. The three highest OR values were found in ≥13 months duration of breastfeeding, the number of children, >10 husband education predictors [OR (95% C.I): 7.9 (1–65.2), 6.9 (2–23.8), 2.1 (0.8–5.5)] for the EBF, respectively. While the highest of the three OR values is found in family size >4 members, underweight infants, and infants age ≥19 months [OR (95% C.I): 7.1 (1.8–28.3); 4.7 (0.4–50.2); 2.7 (0.6–11.5)] for the mixed BF variable.

The effects of the mother's education, father's education, skin-to-skin period, number of children, mother's health, and living region reported an increase in the odds ratio. Additionally, they were more likely to breastfeed exclusively. The odds ratio of the breastfeeding duration and skin-to-skin period showed a significant and negative effect on mixed breastfeeding, indicating an association with a decreased odds ratio of mixed breastfeeding. Furthermore, the results reported a significant and positive impact of having a maid at home, family size, and family income, indicating an association with an increased odds ratio of mixed breastfeeding.

## Discussion

Breastfeeding provides both mothers and infants with great benefits and is highly recommended for all mothers. This study assessed the prevalence of exclusive breastfeeding and the determinant factors influencing exclusive breastfeeding practices among women living in Dubai and Sharjah. The study showed that only 24.4% of women practiced EBF. Despite the WHO recommendations regarding breastfeeding and EBF benefits for both infant growth and reduction in the risk of diseases, women in the UAE are still far from reaching the target WHO goal (1). In comparison, more than half of the women practiced mixed feeding (57.1%) in both Dubai and Sharjah, which is similar to a previous study in the UAE among Emirate women, which reported that only 24% of the participants have exclusively breastfed their infants (21). This indicates that the practices of breastfeeding did not change despite all the national efforts (37).

Our study showed that the main factors associated with women refraining from EBF were being working women and living in Dubai. This was similar to a study conducted in Abu Dhabi, where 60% of the working women stopped breastfeeding (22). In the UAE, the women's labor force increased dramatically between 1990 and 2019 at the rates of 28.9 and 52.39%, respectively (46), and further rose to 57.5 in 2020 according to the World Bank (47), which increased the number of working women in the UAE. Therefore, our results may reflect the barriers faced by working mothers in the UAE, which are deterrents to breastfeeding. This might be because of the number of working hours or duration of maternal leave. In addition to the lack of nurseries in the mother's workplace, making it difficult for working women to breastfeed their infants (22). Previous studies have also reported that maternal employment was negatively associated with exclusive breastfeeding (16, 48–50). However, this study showed that women living in Sharjah were more committed to EBF than those living in Dubai. This may be related to several factors, such as the EBF education and awareness programs in Sharjah (40).

On the other hand, the results showed that women who had a maid at home (70%) were more likely to mixed feed their infants than those who had no maids at home. This might be related to the fact that women stay away for long hours from their infants; it would be much easier for the maid to control the infant's hunger by using formula milk when the mother is away from home. This is consistent with a study conducted in Saudi Arabia on the effect of having a maid on raising children and mothers' attachment. It was found that more than half of the maids were responsible for both household cleaning and nourishing the infants, and were mainly using bottle feeding because it is more convenient and fast, especially when the mother is working and away from home (51).

Our study showed that mothers who did not experience lactation amenorrhea and used contraceptives were more prone to mixed feeding. The relationship between menstruation (ovulation) and breastfeeding has a positive relationship; in that, studies have shown that the more frequent breastfeeding and the duration of breastfeeding, the longer extended period of menstrual cycle stopping among women. Therefore, it is expected that women who do not breastfeed more frequently will have their ovulation sooner than those who adhere to the frequency of breastfeeding. This was found in earlier studies in Bangladesh that resumed the menstrual cycle and mixed feeding, among which the study by Radwan (52).

Early skin-to-skin contact with the newborn after delivery was found in our study to predict EBF practices, similar to that found by Moor et al. (53), as this contact would create an intimate relationship and interaction between and build feelings of warmth, care, and connection. Skin-to-skin contact also enhances the release of oxytocin hormone, which is beneficial for controlling postpartum hemorrhage (53). It was also found in one study by Conde-Agudelo et al. (54) that the Kangaroo strategy of skin-to-skin contact together with exclusive breastfeeding would decrease the infant mortality rate.

The study showed that breastfeeding in the first hour after delivery is highly associated with EBF, in that the production of milk will be initiated, and women would feel more satisfied with their infant needs. While infants might feel attached to breastfeeding and be more connected to their mothers, eventually leading to a longer breastfeeding period and an increased EBF commitment. Early initiation of breastfeeding is highly recommended by the WHO and UNICEF (4). Early initiation was also found to be significantly associated with EBF in our study; the earlier it started, the more committed. Many studies support the importance of initiating breastfeeding and the relationship with EBF (22, 55). In this study, a high income was also found to be strongly associated with mixed feeding. Similarly, in a previous study in UAE, high income was associated with the cessation of breastfeeding (22). High income was found to be associated with cessation of breastfeeding in several other studies worldwide (56, 57).

The study also showed that women who breastfed their infants for more than 6 months and those who currently breastfed their infants were significantly more likely to undergo EBF than the others. This finding was consistent with the results of a study conducted in Cyprus (58). This could be related to the fact that women who feel committed to breastfeeding their infants will be more likely to dedicate themselves to EBF at an early stage; on the other hand, women who choose not to feed for more than 6 months would be more likely not highly dedicated and committed to EBF and prone to mixed feeding.

Among the predictors of mixed feeding, our study showed infant's underweight, mother's education, infant gender, having a maid at home, mother's age at marriage, family size, family income, and living place are significant. Infant underweight is culturally associated with insufficient milk production in the Arab and Gulf countries, or the milk is not very nutritious for the baby; therefore, many mothers tend to go for mixed feeding and eventually, after some time, cessation of breast milk. This is consistent with many studies and has been discussed among several other cultures and Arab cultures (21, 38, 59, 60). It was specifically reported in an early study that women in the UAE initiated mixed feeding as early as the first month of infancy for the same reason (21).

Interestingly, our study did not show a significant association between maternal health problems, method of delivery, and maternal BMI with EBF, although it was previously found to be among the determinants of EBF in the Gulf countries (22, 23, 41). Regarding the mode of delivery, although it showed no significant association with EBF, more than half of the women who delivered *via* cesarean section reported mixed feeding, which is similar to previous studies because of operation pain and discomfort (22, 41, 60). Regarding women's BMI and EBF, it was found previously that maternal obesity was considered a risk factor for initiating breastfeeding among women in developing countries (61). Since the physiological and psychological determinants among obese women prevent them from initiating breastfeeding, neither sustain the practice for a longer period, looking to the fact that prolactin production is lower, big breasts with large areola and inverted nipples make breastfeeding difficult (62). Our study showed that approximately 51% of the obese and overweight participants fed their children mixed feeding, compared to 22% who practiced EBF. This could tell you that despite the difficulties that weight can endorse, those who are willing to feed their children mother's milk were more committed as in the Arab culture the obese women have more nutritional milk than underweight women, and therefore family might support breastfeeding.

Some limitations of this study should be considered when interpreting its results. First, the data in this study represent two out of the seven UAE emirates. Although Dubai and Sharjah are two of the most densely populated emirates, geographical differences may exist when all seven emirates are considered. Another limitation was the small number of participants who met the study criteria for selection and missing data for some variables. Therefore, the results are not generalizable to the entire UAE, and additional research to cover all the seven emirates is needed. Another limitation of this study was the recall bias. Recall bias was common among the participants who were interviewed about past events.

## Conclusion

This study has highlighted several important findings. Mothers who were not working and those living in Sharjah had a higher prevalence of EBF. Other factors associated with EBF were early skin-to-skin contact and breastfeeding during the first-hour post-delivery. Further research to cover all the other seven emirates and determinant factors for EBF is recommended to encourage breastfeeding-supportive working environment policies, a health promotion program for exclusive breastfeeding during antenatal health visits, together with initiating health policies in maternal hospitals to encourage the initiation of breastfeeding during the first hour of birth and the introduction of skin-to-skin contact during the first 5 min of birth is highly recommended. Future studies regarding the effect of EBF on infant growth and development in the UAE are highly encouraged.

## Data Availability Statement

The raw data supporting the conclusions of this article will be made available by the authors, without undue reservation.

## Ethics Statement

The studies involving human participants were reviewed and approved by Both Zayed University Ethical Committee and the UAE Ministry of Health Ethical Committee approved this study. The patients/participants provided their written informed consent to participate in this study.

## Author Contributions

HA designed the study and HA and HR recruited the participants and supervised the data collection. HA and RQ analyzed the data. HA, EA, and ZT wrote the manuscript. HA designed the study and manuscript writing. HR reviewed the manuscript. All contributed authors of this original manuscript authorized the final version of the manuscript. All authors read and approved the final version of the manuscript.

## Funding

The study was funded by the Research Office at Zayed University (R16050).

## Conflict of Interest

The authors declare that the research was conducted in the absence of any commercial or financial relationships that could be construed as a potential conflict of interest.

## Publisher's Note

All claims expressed in this article are solely those of the authors and do not necessarily represent those of their affiliated organizations, or those of the publisher, the editors and the reviewers. Any product that may be evaluated in this article, or claim that may be made by its manufacturer, is not guaranteed or endorsed by the publisher.
